# Reduced brain volume and white matter alterations in *Shank3*‐deficient rats

**DOI:** 10.1002/aur.2568

**Published:** 2021-07-27

**Authors:** Carla E. M. Golden, Victoria X. Wang, Hala Harony‐Nicolas, Patrick R. Hof, Joseph D. Buxbaum

**Affiliations:** ^1^ Department of Psychiatry Icahn School of Medicine at Mount Sinai New York New York USA; ^2^ Seaver Autism Center for Research and Treatment Icahn School of Medicine at Mount Sinai New York New York USA; ^3^ BioMedical Engineering and Imaging Institute Icahn School of Medicine at Mount Sinai New York New York USA; ^4^ Nash Family Department of Neuroscience Icahn School of Medicine at Mount Sinai New York New York USA; ^5^ Friedman Brain Institute Icahn School of Medicine at Mount Sinai New York New York USA; ^6^ Mindich Child Health and Development Institute Icahn School of Medicine at Mount Sinai New York New York USA; ^7^ Department of Genetics and Genomic Sciences Icahn School of Medicine at Mount Sinai New York New York USA; ^8^ Present address: Center for Neural Science New York University New York NY USA

**Keywords:** autism spectrum disorder, diffusion tensor imaging, magnetic resonance imaging, Shank3

## Abstract

Mutations and deletions in the *SHANK3* gene cause the major neurodevelopmental features of Phelan–McDermid syndrome (PMS), which is characterized by intellectual disability, autism spectrum disorder, and sensory hyporeactivity. *SHANK3* encodes a key structural component of excitatory synapses important for synaptogenesis. Clinical assessments and limited brain imaging studies of patients with PMS have uncovered regional volume reductions and white matter thinning. While these impairments have been replicated ex vivo in pups of a rat model, brain structure has not been assessed in rats in vivo or in adults. We assessed the brain structure of heterozygous and homozygous adult *Shank3*‐deficient male rats in comparison to wild‐type littermates with magnetic resonance imaging using both anatomical assessments and diffusion tensor imaging (DTI). *Shank3*‐deficient rats showed a reduction in overall brain size and the absolute volume of the neocortex, piriform cortex, thalamus, forebrain, inferior and superior colliculi, internal capsule, and anterior commissure. The superior colliculus was decreased in relative volume. DTI revealed that axial diffusion and fractional anisotropy were reduced in the external capsule and mean diffusion was increased in the fornix, suggesting that restriction of diffusion perpendicular to the axis of the axonal fibers was impaired in these white matter tracts. Therefore, *Shank3*‐deficient rats replicate the reduced brain volume and altered white matter phenotypes present in PMS. Our results indicate that the loss of a glutamatergic synaptic protein, Shank3, has structural consequences at the level of the whole brain. The brain regions that were altered represent potential cross‐species structural biomarkers that warrant further study.

## Lay Summary

Phelan–McDermid syndrome can cause brain abnormalities. To investigate whether these could be replicated in a rat model, we assessed brain structure in rats lacking Shank3, the synaptic protein that is depleted in Phelan–McDermid syndrome. We found that Shank3‐deficient rats have smaller brains than littermate controls and deficits in their white matter pathways, similar to what is observed in people with Phelan–McDermid syndrome. This rat model can therefore be used in follow‐up studies to determine how a loss of Shank3 can lead to alterations in brain structure. Furthermore, our results indicate that this singular synaptic protein is important for the development of the whole brain.

## INTRODUCTION

Phelan–McDermid syndrome (PMS) is a rare neurodevelopmental disorder caused by deletions in or a rearrangement of the terminal chromosomal region 22q13.3 that encompasses the *SHANK3* gene or by a pathogenic deletion or mutation within *SHANK3* (Harony‐Nicolas et al., 2015). The characteristics of PMS include intellectual disability, autism spectrum disorder (ASD), attention deficits, motor abnormalities, seizures, and macrocephaly. Alterations in reactivity to sensory stimuli are frequently observed (De Rubeis et al., 2018; Droogmans et al., 2020). Understanding how the pathology that results from *Shank3* deficiency affects brain structure in PMS pathogenesis could provide biomarkers to focus on in patients and rodent models. Neuroimaging studies using small cohorts of patients have shown that individuals with PMS have reduced volumes of structures in the basal ganglia and cerebellum (Aldinger et al., 2013; Srivastava et al., 2019), white matter thinning (Philippe et al., 2008; Soorya et al., 2013), and altered fiber tracts (Bassell et al., 2020; Jesse et al., 2020). Brain structure has not yet been explored in vivo or in an adult rat model of PMS. In this study, we used adult heterozygous and homozygous knockout (KO) male rat models to investigate the effect of *Shank3* deficiency on brain structure with magnetic resonance imaging in vivo because we previously demonstrated that they have deficits in social memory, attention, and synaptic plasticity (Harony‐Nicolas et al., 2017).

## METHODS

### Animal care and husbandry

We used 3‐month‐old male wild‐type (WT), *Shank3* ^*+/−*^, and *Shank3* ^*−/−*^ littermate rats. *Shank3*‐deficient rats were generated using zinc‐finger nucleases on the outbred Sprague–Dawley background, as previously described (Harony‐Nicolas et al., 2017). Rats were kept under veterinary supervision in a 12‐h reverse light/dark cycle at 22 ± 2°C. Animals were pair‐caged with food and water available ad libitum. All animal procedures were approved by the Institutional Animal Care and Use Committee at the Icahn School of Medicine at Mount Sinai.

### MRI

All imaging was performed using a Bruker Biospec 70/30 7 T scanner with a B‐GA12S gradient insert (gradient strength 440 mT/m and slew rate 3444 T/m/s). A Bruker 4‐channel rat brain phased array was used for all data acquisition in conjunction with a Bruker volume transmit 86‐cm coil. All rats were imaged on a heated bed and respiration was monitored continuously until the end of the scan. The animals were anesthetized with isoflurane (3% induction and 1.5% maintenance). After a three‐plane localizer, a field map was acquired and the rat brain was shimmed using Mapshim software. A DTI protocol was acquired with a pulsed gradient spin echo—echo‐planar imaging sequence with the following parameters: repetition time (TR) = 5000 ms, echo time (TE) = 22.6 ms, 4 segments, 30 gradient directions with *b* value = 1000 s/mm^2^ and 5 B0's, field of view (FOV) = 25 mm, matrix = 128 × 128, slice thickness = 1 mm, skip = 0, 6 averages, and an acquisition time of 1 h. The voxel size was 0.195 × 0.195 × 1 mm^3^ (1000 μm‐thick). A high resolution T2 anatomical scan was obtained with a 3D rapid acquisition with relaxation enhancement (RARE) sequence with a RARE factor of 8, TR = 777 ms, effective TE = 52 ms, FOV = 30 mm × 27.25 mm × 30 mm, and a matrix size of 256 × 256 × 128. The voxel size was 0.117 × 0.117 × 0.234 mm^3^ (234 μm‐thick).

### MRI analytical pipeline with manual editing

An MRI processing pipeline was used to perform semi‐automated nonbiased brain segmentation, while blinded to genotype (WT: *N* = 6, *Shank3* ^*+/−*^: *N* = 10, *Shank3* ^*−/−*^: *N* = 10) (Budin et al., 2013). There were six steps: rigid registration of images to each other, generation of a whole‐brain mask per image, averaging of all images, creation of a whole‐brain mask for the averaged image, segmentation of the average mask by regions of interest (ROIs), parcellation propagation of the segmented mask to individual subjects, and computation of ROI‐based statistics for individual images. The deformation necessary to warp each subject's image to the average was used to calculate ROI volume. After each mask was generated, it was improved manually in ITK‐SNAP (www.itksnap.org). The segmentation into ROIs was determined by a template that was hand‐segmented into 32 brain regions, listed in Table S1.

Automatically generated segmented masks for individual images that did not match the average segmented mask (two WT and one *Shank3* ^*−/−*^ T2 mask) and individual data points that were outside of 1.5 times the interquartile range, were excluded from the analysis. Whole‐brain masks were used to determine whole‐brain measurements. Volumes were calculated from T2 images and mean voxel intensity was measured in both T2 and DTI images. Only white matter structures were included in the DTI analysis (Table S1).

### Statistics

Genotype was the only between‐groups factor. The distribution of each dependent variable was assessed with a Shapiro–Wilk's test. If the distribution was normal, a two‐way analysis of variance (ANOVA) was used. If it was nonparametric, a Kruskal–Wallis test was performed. We corrected for the multiple comparisons made across ROIs, including the whole brain as an ROI, with a Bonferroni correction. Pairwise comparisons were made for main effects with nominal *p* values <0.05. A Tukey HSD test was used for parametric pairwise comparisons and a Dunn's test was used for nonparametric. An adjustment of the *p* values was made to account for the additional comparisons and reported below. The effect size of each volumetric change was measured with a Cohen's *d*. Custom scripts written in the R statistical programming environment were used for these analyses (R Core Team, 2020).

## RESULTS

### Shank3 *deficiency results in reduced brain volume*

**FIGURE 1:**
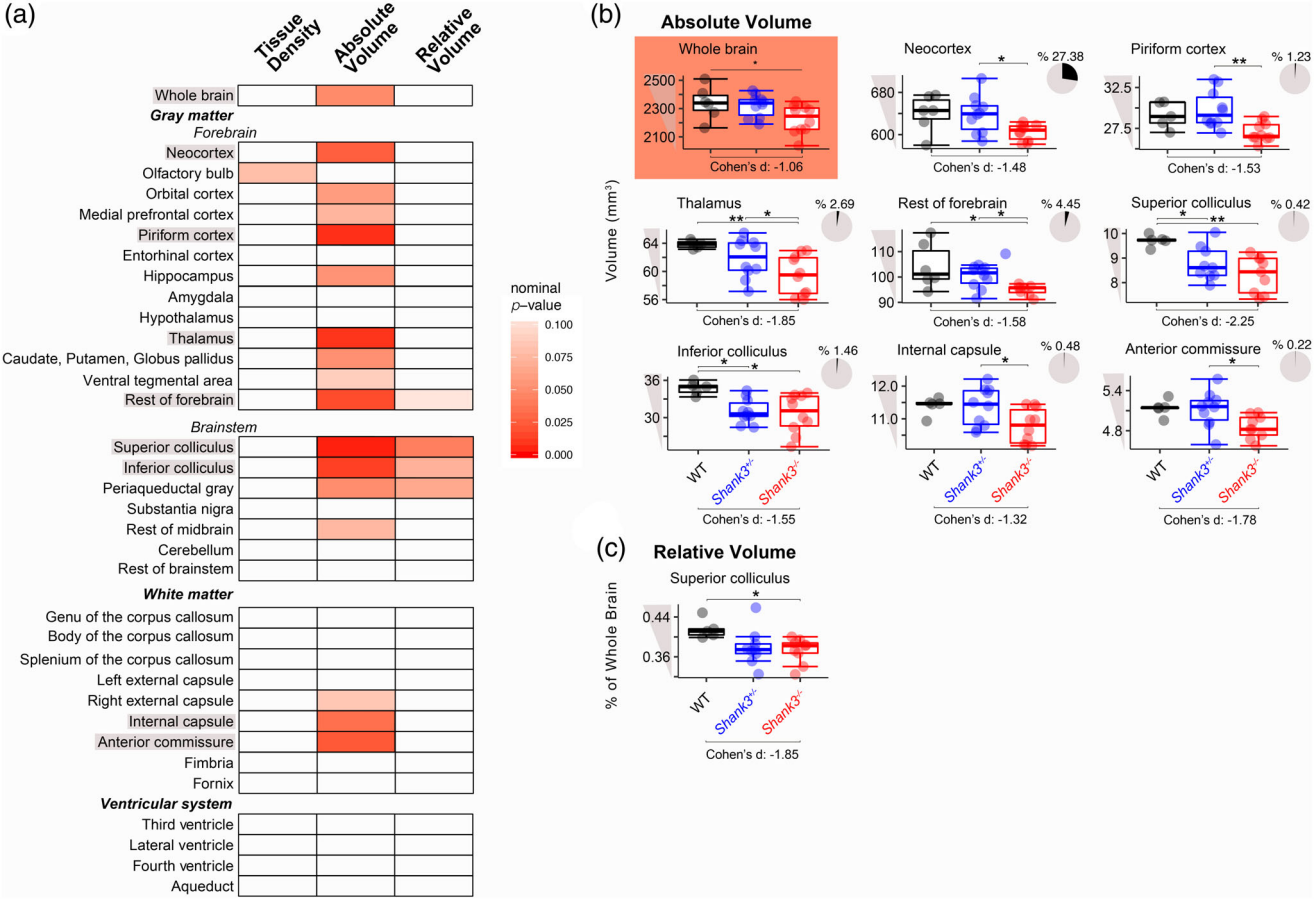
Tissue density and absolute and relative volume of brain regions of wild‐type (WT) and *Shank3*‐deficient littermates using T2 magnetic resonance imaging (MRI). (a) Heatmap of *p* values from analysis of variance (ANOVA) and Kruskal–Wallis tests for an effect of genotype on tissue density and absolute and relative volumes where an increase in red denotes an increase in significance of nominal *p* values. Boxplots of the brain regions that had nominal *p* values <0.05 when (c) absolute and (d) relative volume were compared. Effect sizes for the differences between WT and *Shank3* ^−/−^ littermates were reported as Cohen's *d* below the graphs and are considered large if they are >|0.8|. The pie charts for each absolute volume represent the percentage of the total volume that each region comprises in the WT rats. Significance bars represent the effect of genotype or pair‐wise comparisons (Tukey HSD or Dunn's test) that were performed following a nominal *p* value for the effect of genotype that was <0.05, (WT: *N* = 6, *Shank3* ^+/−^: *N* = 10, *Shank3* ^−/−^: *N* = 10), **nominal *p* for pair‐wise comparison <0.01, *nominal *p* for pair‐wise comparison <0.05

Structural T2‐weighted MRI was used to assess both ROI tissue density and volume in *Shank3* ^*−/−*^, *Shank3* ^*+/−*^, and WT littermates (Table S2). None of the nominal *p* values for the effect of genotype or the pairwise comparisons survived a Bonferroni correction. However, there was a trending effect of genotype on whole brain volume, where the *Shank3* ^*−/−*^ rats had a smaller brain size than their *Shank3* ^*+/−*^ and WT littermates (Figure 1a,b; ANOVA: *p =* 0.049). This reduction in overall brain volume was driven by trending decreases in the absolute volumes of larger gray matter brain structures in *Shank3* ^*−/−*^ rats, including the neocortex, piriform cortex, thalamus, and the rest of the forebrain (regions of the forebrain not otherwise included as ROIs; Figure 1a,b; ANOVA followed by Tukey HSD: *Shank3* ^*+/−*^ vs. *Shank3* ^*−/−*^: *p adj. =* 0.043; ANOVA followed by Tukey HSD: *Shank3* ^*+/−*^ vs. *Shank3* ^*−/−*^: *p adj. =* 0.0065; Kruskal–Wallis followed by Dunn's test: WT vs. *Shank3* ^*−/−*^: *p adj*. = 0.0056, *Shank3* ^*+/−*^ vs. *Shank3* ^*−/−*^: *p adj*. = 0.02; Kruskal–Wallis followed by Dunn's test: WT vs. *Shank3* ^*−/−*^: *p adj*. = 0.018, *Shank3* ^*+/−*^ vs. *Shank3* ^*−/−*^: *p adj*. = 0.012). The inferior and superior colliculi also had trending reductions in absolute volume in *Shank3‐*deficient rats compared to WT littermates (Figure 1a,b; ANOVA followed by Tukey HSD: WT vs. *Shank3* ^*−/−*^: *p adj*. = 0.013, WT vs. *Shank3* ^*+/−*^: *p adj. =* 0.027 and ANOVA followed by Tukey HSD: WT vs. *Shank3* ^*−/−*^: *p adj*. = 0.0027, WT vs. *Shank3* ^*+/−*^: *p adj. =* 0.043). Furthermore, two white matter structures, the internal capsule and anterior commissure, had trending reductions in absolute volume in *Shank3* ^*−/−*^ rats (Figure 1a,b; ANOVA followed by Tukey HSD: WT vs. *Shank3* ^*−/−*^: *p adj*. = 0.065, *Shank3* ^*+/−*^ vs. *Shank3* ^*−/−*^: *p adj. =* 0.035 and WT vs. *Shank3* ^*−/−*^: *p adj*. = 0.099, *Shank3* ^*+/−*^ vs. *Shank3* ^*−/−*^: *p adj. =* 0.049). The effect sizes of all changes were large (Cohen's *d* > |0.8|). Tissue density was not altered (all nominal *p* values were >0.05) and no alterations were identified in the volume of the ventricular system.

We next examined relative volume by calculating the percentage of total brain volume. Given that the entire *Shank3* ^*−/−*^ rat brain was smaller, many of the ROIs that were decreased in absolute volume were not also decreased in relative volume. The only region that had a trending reduction in relative volume in *Shank3* ^*−/−*^ rats was the superior colliculus (Figure 1a,c; ANOVA followed by Tukey HSD: WT vs. *Shank3* ^*−/−*^: *p adj*. = 0.041).

### *Loss of* Shank3 *leads to deficits in white matter region integrity*

**FIGURE 2:**
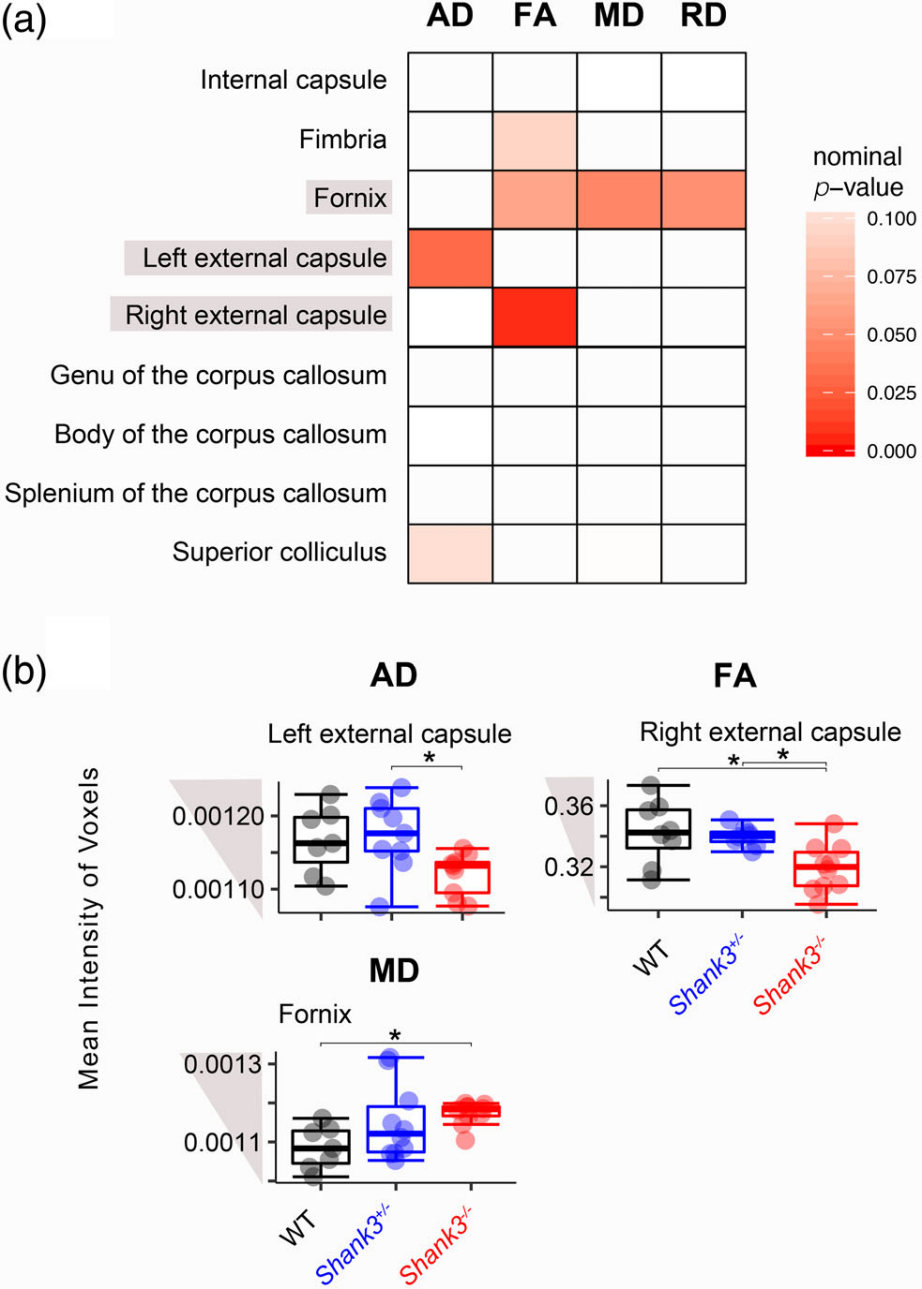
Changes in diffusion indices of wild‐type (WT) and *Shank3*‐deficient littermates using diffusion tensor imaging (DTI). (a) Heatmap of *p* values from analysis of variance (ANOVA) and Kruskal–Wallis tests for the effect of genotype on axial diffusion (AD), fractional anisotropy (FA), mean diffusion (MD), and radial diffusion (RD), where an increase in red denotes increased significance. (b) Boxplots of mean intensity of voxels across altered regions in *Shank3*‐deficient rats. Significance bars represent pair‐wise comparisons from a Tukey HSD test that was performed following a nominal *p* value for the effect of genotype that was <0.05, (WT: *N* = 6, *Shank3* ^+/−^: *N* = 10, *Shank3* ^−/−^: *N* = 10), *nominal *p* for pair‐wise comparison <0.05

We used DTI to assess the integrity of nine white matter pathways in *Shank3*‐deficient rats and WT littermates (Table S2). Four indices were calculated and used to describe the diffusion: axial, radial, and mean diffusion (AD, RD, and MD) and fractional anisotropy (FA). None of the nominal *p* values survived a Bonferroni correction. However, there was a trending reduction in two parallel measures in the external capsule of *Shank3* ^*−/−*^ rats: AD in the left and FA in the right hemisphere (Figure 2; ANOVA followed by Tukey HSD: *Shank3* ^*+/−*^ vs. *Shank3* ^*−/−*^: *p adj. =* 0.037; ANOVA followed by Tukey HSD: WT vs. *Shank3* ^*−/−*^: *p adj*. = 0.011, *Shank3* ^*+/−*^ vs. *Shank3* ^*−/−*^: *p adj*. = 0.026). MD and RD (not shown) had trending increases in the fornix of *Shank3* ^*−/−*^ rats compared to WT (Figure 2; ANOVA followed by Tukey HSD: WT vs. *Shank3* ^*−/−*^: *p* = 0.04, *p* = 0.052).

## DISCUSSION

In this study, we uncovered structural perturbations in the brains of *Shank3*‐deficient rats, including an overall reduction in brain volume driven by a decrease in the volume of large, gray matter regions, and alterations in the structural integrity of white matter domains. Both phenotypes have been identified in humans and mice with *Shank3* deficiency (Aldinger et al., 2013; Bassell et al., 2020; Berg et al., 2018; Jesse et al., 2020; Philippe et al., 2008; Schoen et al., 2019; Soorya et al., 2013; Srivastava et al., 2019). Our work also complements findings from an ex vivo study of *Shank3*‐deficient pups that identified trending decreases in regional volumes of the basal ganglia and hypothalamus (Berg et al., 2018). This indicates that reduced brain volume and altered white matter are potential cross‐species structural biomarkers that warrant further study. In future work, the brains of juvenile and female *Shank3*‐deficient rats should be analyzed.

The white matter deficits we identified in *Shank3*‐deficient rats included a decrease in absolute volume of the internal capsule and anterior commissure, a reduction in AD and FA in the external capsule, and an increase in MD and RD in the fornix. This suggests that white matter microstructure is not properly able to restrict diffusion to follow along the axis of the axonal fibers in the external capsule and fornix. This could affect the function of corticocortical and corticosubcortical projections. The fornix is one of the major output pathways of the hippocampus, where both long‐term potentiation and mGluR‐dependent long‐term depression is impaired in these rats (Harony‐Nicolas et al., 2017), further indicating that *Shank3* deficiency likely leads to white matter disease.

Three of the regions that were reduced in volume, the superior and inferior colliculi and the thalamus, are involved in sensory processing, suggesting a potential locus for the sensory deficits often documented in PMS and the rat model (De Rubeis et al., 2018; Droogmans et al., 2020; Engineer et al., 2018). Interestingly, we recently reported similar structural alterations in another rat model of a monogenic cause of ASD, Fragile X syndrome, using the same imaging techniques and parameters (Golden et al., 2020), establishing the potential for commonality across syndromes. The relationship between the structure of these regions and their functional consequences should be further investigated in rodent models of ASD.

Importantly, these findings also show that the synaptic structural protein encoded by the *Shank3* gene, Shank3, is necessary for the male rat brain to develop to a normal size. This is consistent with prior work at the cellular level that has shown that *Shank3* deficiency in *Shank3* ^*e4–9*^, *Shank3B* ^*−/−*^, and *Shank3* ^*+/−*^ mice leads to decreased expression of synaptic proteins, smaller glutamatergic postsynaptic densities, higher numbers of perforated hippocampal synapses, reduced dendritic arborizations and length, and decreased spine density in medium spiny neurons (Peça et al., 2011; Uppal et al., 2015; Wang et al., 2011). Future studies should examine how these cellular phenotypes affect the structure of the brain regions we found to be reduced.

## Supporting information


**Table S1** Regions of interest (ROIs) used for segmentation of images.

**Table S2** Statistical output for all analyses.
